# Modulation of the Arginase Pathway in the Context of Microbial Pathogenesis: A Metabolic Enzyme Moonlighting as an Immune Modulator

**DOI:** 10.1371/journal.ppat.1000899

**Published:** 2010-06-17

**Authors:** Priyanka Das, Amit Lahiri, Ayan Lahiri, Dipshikha Chakravortty

**Affiliations:** Center for Infectious Disease Research and Biosafety Laboratories, Department of Microbiology and Cell Biology, Indian Institute of Science, Bangalore, India; University of California San Diego, United States of America

## Abstract

Arginine is a crucial amino acid that serves to modulate the cellular immune response during infection. Arginine is also a common substrate for both inducible nitric oxide synthase (iNOS) and arginase. The generation of nitric oxide from arginine is responsible for efficient immune response and cytotoxicity of host cells to kill the invading pathogens. On the other hand, the conversion of arginine to ornithine and urea via the arginase pathway can support the growth of bacterial and parasitic pathogens. The competition between iNOS and arginase for arginine can thus contribute to the outcome of several parasitic and bacterial infections. There are two isoforms of vertebrate arginase, both of which catalyze the conversion of arginine to ornithine and urea, but they differ with regard to tissue distribution and subcellular localization. In the case of infection with *Mycobacterium, Leishmania*, *Trypanosoma*, *Helicobacter*, *Schistosoma*, and *Salmonella spp*., arginase isoforms have been shown to modulate the pathology of infection by various means. Despite the existence of a considerable body of evidence about mammalian arginine metabolism and its role in immunology, the critical choice to divert the host arginine pool by pathogenic organisms as a survival strategy is still a mystery in infection biology.

## Introduction

Arginase, the arginine hydrolytic enzyme, was first discovered by Kossel and Dakin in 1904 in the mammalian liver [1]. It is a binuclear manganese metalloenzyme that catalyzes the hydrolysis of L-arginine to urea and ornithine. There are two isoforms of the enzyme, namely arginase I and II. Arginase I is a trimeric cytosolic protein, total size 34,700 Da, and is expressed in erythrocytes in humans and higher primates. The second isoform, arginase II, is also a trimeric mitochondrial protein, total size 36,100 Da, and is expressed in extrahepatic tissues like the small intestine, kidney, brain, monocytes, and macrophages [2]. Arginase II is synthesized as a pre-protein, imported to mitochondria, and processed to the mature form [3], [4]. In addition, some pathogens possess their own arginase, which is required to produce endogenous urea [5], [6].

One of the competing enzymes of arginase for L-arginine is nitric oxide synthase (NOS). There are three types of nitric oxide synthases, namely, inducible NOS (iNOS), neuronal NOS, and endothelial NOS. iNOS is not constitutively expressed but highly induced by lipopolysaccharide (LPS), lipoteichoic acid (LTA), and Type 1 cytokines like interferon gamma (IFNγ), tumour necrosis factor alpha (TNF-α), interleukin 1 (IL-1), and IL-2. Nitric oxide (NO) contributes to the innumerable physiological processes, the understanding of which is relevant to fathom the pathogenesis of infection [3], [7]. NO is the central component of innate immunity in murine macrophages and is an effective antimicrobial agent, especially against intracellular pathogens such as *Mycobacterium tuberculosis*, *Leishmania major*, *Salmonella*, and also against extracellular bacteria like *Escherichia coli* [8].

**Figure 1:**
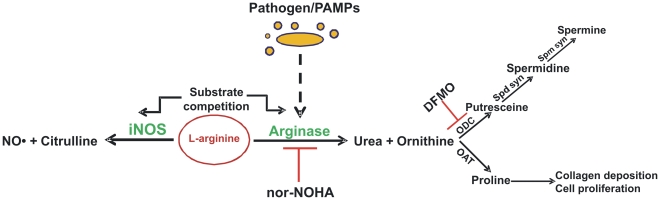
Overview of mammalian arginine metabolism. Only enzymes that directly use or produce arginine, ornithine, or citrulline are identified, and not all reactants and products are shown. Inhibition of specific enzymes is indicated by ⊥. DFMO, difluoromethyl ornithine; iNOS, inducible nitric oxide synthase; NO, nitric oxide; nor-NOHA, nor-N^ω^-hydroxy-l-arginine; OAT, ornithine aminotransferase; ODC, ornithine decarboxylase; PAMPs, pathogen-associated molecular patterns; Spd Syn, spermidine synthase; Spm Syn, spermine synthase.

It has been proved that the availability of the intracellular arginine is a rate-limiting factor in NO synthesis, although extracellular arginine concentration has been shown to play a more important role in regulating NO synthesis compared to intracellular arginine [3]. Arginase and NOS use arginine as a common substrate and compete with each other for this substrate. Although Km of arginase is in mM range and of NOS in µM range, arginase Vmax at body pH is 1,000 times more than that of NOS, indicating that similar rates of substrate usage occur for both enzymes at a low arginine concentration [3]. Interestingly, cAMP, LPS, and Type 2 cytokines such as IL-4, IL-13, and TGF-β induce arginase I expression in macrophages. Type 1 cytokines like IFNγ increase NO production by NOS2 induction and inhibit IL-4- and IL-10-driven arginase I activity. The alternative activation by Type 2 cytokines like IL-4 and IL-13 inhibits NOS2 function and induces arginase I, leading to increased humoral immunity, tissue repair, and allergic and anti-parasitic response [9]. In addition, the polyamines produced in the arginase pathway downregulate pro-inflammatory cytokine release. Arginase activation results in collagen synthesis by proline and therefore is hypothesized to be required in wound healing [10]. The different players and the critical regulation of the arginase isoforms is depicted in Figure 1.

Hence, the critical interplay between arginase and NOS might regulate the outcome of several pathologic conditions by modulating the amount of NO produced. Furthermore, the production of polyamines and other metabolic intermediates of the arginase pathway could also dictate the severity of any infection. On the other hand, the cytokine profile after any infection is a key regulator of both iNOS and arginase induction and thus determines the disease outcome. To date, sufficient evidence has accumulated to conclude that arginase is not a mere urea cycle enzyme; rather, it is a moonlighting enzyme that acts as a double-edged sword in immunity. This review considers the recent studies that deal with the modulation of expression and function of the arginase isoforms by various successful pathogens.

## Modulation of the Arginase Pathway by Various Pathogens

### Bacterial Interference of Arginase

*Helicobacter pylori* is a Gram-negative micro-aerophillic bacteria that selectively colonize the human stomach. It causes chronic gastritis, peptic ulcer, gastric carcinoma, and lymphoma, leading to its classification as a class I carcinogen [11]. *H. pylori* arginase is encoded by the gene *rocF*, which is constitutively expressed. Although both the wild-type and *rocF* mutant stimulate similar levels of iNOS mRNA, a significantly greater amount of NO production is elicited by the mutant strain in RAW macrophages at a physiologically relevant arginine concentration. This agrees with the hampered survival of the *rocF* mutant strain, thus indicating the role of the pathogen-induced arginase to quench arginine from iNOS [12], [13]. In a previous study, the *Helicobacter* arginase was found to protect the bacteria from acid stress, and an arginase-deficient strain showed attenuated colonization in the mouse model [5]. In addition, *Helicobacter* arginase also impairs host T cell function by reducing CD3ζ chain expression, and this phenotype might play a very important role during *Helicobacter* infection [14]. These unique functions imply that the *Helicobacter* arginase has evolved to allow the bacteria to effectively compete with its host in the mucous layer. The decrease in host NO production by *Helicobacter* arginase may have two important outcomes. The first one certainly is to avoid host nitrosative stress, and the second one might be to reduce the NO-mediated damage of the gastric mucosa. If the second possibility is true, then arginase must be one of the very essential factors for the long-term survival and proliferation of this pathogen in the gastric niche.

In addition, *H. pylori* upregulates the host arginase II in RAW macrophages and in mouse and human gastritis tissues and induces apoptosis. The product of the arginase pathway, ornithine, is acted upon by ornithine decarboxylase (ODC) to generate ornithine. *H. pylori*–mediated apoptosis was blocked in the presence of the host arginase inhibitor *N* ^ώ^-hydroxy-nor-L-arginine (nor-NOHA), and also in the presence of an ODC inhibitor, α-difluoromethylornithine (DFMO). iNOS inhibition had no effect on *H. pylori*–mediated apoptosis. Arginase II induction was not observed in mice infected with the nonpathogenic *E. coli* DH5α strain [15]. This clearly demonstrates that the induction of host arginase is directly correlated with the virulence of the organism. However, there are no reports of arginase induction in hosts with pathogenic *E. coli*, indicating the specific phenotype varies from pathogen to pathogen. Further, *E. coli* lacks endogenous arginase. Ornithine is converted to the polyamine spermidine by spermidine synthase (Spd Syn). The next step of polyamine production consists of the production of another polyamine spermine by action of the spermine synthase (Spm Syn) enzyme (Figure 1). *H. pylori* can utilize this spermine to restrain immune response in the activated macrophages by inhibiting pro-inflammatory gene expression. Spermine could also prevent the antimicrobial effects of NO by inhibiting iNOS translation in the macrophages infected by *H. pylori* [16]. Future work should provide insights into whether the nor-NOHA-mediated host arginase inhibition can also decrease *H. pylori* survival in human gastric tissues.

Another intracellular pathogen, *Salmonella enterica* serovar Typhimurium, also utilizes the host arginase for its own survival inside mouse macrophages. *Salmonella* is capable of causing clinical signs that range from self-limiting diarrhea to severe fibrinopurulent necrotizing enteritis and life-threatening systemic disease in various hosts. In the spleen of *Salmonella* Typhimurium–infected mice, a clear increase in the arginase II protein level and activity is observed. Inhibition of arginase via nor-NOHA treatment leads to increased NO production and decreased bacterial burden in the secondary lymphoid organs of the infected mice. Arginase induction is dependent on LPS or any other surface pathogen-associated molecular pattern of *Salmonella* [17]. The factor that is of importance here is the side effects of inhibition of the host urea cycle. Further, the specific response of the human pathogen *Salmonella enterica* serovar Typhimurium with respect to arginase induction and modulation of the host arginine metabolism also demands careful consideration.

The successful replication and survival of *M. tuberculosis*, the causative agent of tuberculosis, inside host macrophages depends on the intelligent strategies employed by the bacteria. In the case of *Mycobacterium bovis* (BCG) infection, it has been demonstrated that host urea production slowly increases with the infection time. After 24 hours and 72 hours of infection, there was a significant induction in J774.1 macrophage arginase activity. However, the replication of intracellular BCG increased when arginase activity was inhibited. Hence, it is indicative that the enhanced growth of BCG might be due to increased availability of the intracellular arginine pool to the bacteria in the arginase-blocked condition [18].

In an interesting report, the exact mechanism of arginase induction upon mycobacteria infection was documented. In primary mouse macrophages, the BCG infection–mediated increase in the arginase I protein was shown to be MyD88 and TLR2 dependent and independent of the T helper type 2–associated STAT6 pathway. Deletion of arginase I leads to an enhanced NO response with BCG infection. Supporting the BCG data, an increased arginase I mRNA expression was seen in the lungs of *M. tuberculosis*–infected mice as well. A lower *M. tuberculosis* load was observed in the Arg-1-deficient mice. In the same report it was observed that the liver granuloma from BCG-infected mice produced greater bacterial nitrotyrosine when host arginase was knocked out [19]. The high NO in the Arg-1-deficient mice in response to the *M. tuberculosis* infection in conjunction with superoxide leads to the formation of the highly toxic peroxynitrite. Bacterial nitrotyrosine is formed when peroxynitrite nitrosylates tyrosine residues in bacteria. Although in the J774 macrophage cell line arginase inhibition promoted BCG growth, in the mouse model an opposite phenotype was observed. Arginase I–deficient mice were more efficient at clearing both *M. tuberculosis* and BCG by suppressing NO production from infected macrophages. Recently, in another report it was observed that the supernatant of BCG-infected wild-type macrophages induces arginase I in MyD88^−/−^ macrophages in a STAT3-dependent manner. This induction was attributed to IL-6/IL-10 secretion by the BCG-infected macrophages that induce arginase in neighboring macrophages by autocrine/paracrine IL-6/IL-10 activation of STAT3 [20]. It can be inferred from this that mycobacteria condition uninfected neighboring cells for low NO production by inducing arginase I.

### Modulation of Arginase by Parasites

*Trypanosoma cruzi* is an obligate intracellular protozoan parasite that grows abundantly in the heart and other organs of patients with acute Chagas disease. Cruzipain (Cz), a major *T. cruzi* antigen, was found to increase urea production in splenic macrophages [21]. In a subsequent study, cardiocytes were cultured with Cz and a significant increase in host arginase II was observed. This enhanced arginase in turn promotes survival of the mouse cardiomyocyte [22]. During in vivo infection of *T. cruzi* in BALB/c mice, both arginase I and arginase II were induced in heart tissues. On the one hand, this induction could provide polyamine for the parasite's growth, and on the other might also downregulate the detrimental effects triggered by iNOS in the heart during infection [23]. The reduced apoptosis rate of the cardiomyocyte should ultimately generate an adequate environment for the parasite's growth and dissemination from the host heart. Hence, in *T. cruzi* infection, anti-apoptotic activity of arginase and its downstream enzymes are utilized by the pathogen for survival. We can speculate that NOHA-mediated arginase inhibition might increase this apoptosis and consequently decrease *T. cruzi* survival. This is in contrast to *H. pylori* infection, where there is a host arginase II–dependent polyamine-mediated increase in apoptosis, which might be explained by the differential cell and tissue types wherein polyamine-mediated apoptosis is taking place.

*Trypanosoma brucei* causes trypanosomiasis in both humans and animals in Africa. Both innate and adaptive immune responses of the host are involved in resistance. Moreover, arginase is also induced in *T. brucei* infection [24]. In the susceptible mouse strain BALB/c, arginase I and II mRNA and arginase activity were higher compared to the resistant mouse strain C57BL/6. Both of these phenotypes were directly induced by *T. brucei* infection. In accordance with this phenotype, NO production was significantly higher in the C57BL/6 mice and must be the cause for their resistance. Hence, the macrophage arginase inhibits NO-dependent trypanosome killing. NO generation and parasite survival was restored to the same level in both the susceptible and resistant strains on arginase inhibition. Thus, here arginase serves as a host marker for susceptibility to trypanosome infection [25]. Polyamines are further essential for trypanosome development, as they help in the synthesis of DNA and trypanothione. In protozoans such as *Trypanosoma*, polyamines like spermidine make trypanothione, which is an antioxidant and is required for parasitic proliferation [26], [27]. NOHA treatment decreased parasitic burden in the macrophage population [28]. This was achieved by an increase in arginine availability for iNOS, and it has been observed that supplementation of arginine restores NO-dependent parasite killing. At this point, the use of the *T. brucei*–mediated arginase pathway has been identified, but how the pathogen elicits arginase remains ambiguous.

Curiously, *T. brucei* does not use the arginase pathway for its spread like *T. cruzi*. This contrast might be explained by the fact that in the case of *T. cruzi* infection, apoptosis of the infected heart tissue cells is very much required for the pathogen to invade other organs, which is not the case for *T. brucei*, as it can spread via infected macrophages in the peritoneum cavity. Further investigation is required to determine the effect of arginase at the systemic level during experimental trypanosomiasis.

The protozoan *Leishmania* is an intracellular parasite of mammalian macrophages. To reside successfully in the very cells responsible for its clearance, *Leishmania* requires a bag full of immune evasion tricks. This involves avoidance of phagolysosomal fusion and prevention of activation of acquired immune mechanisms. Macrophages can control *Leishmania* infection when a T helper Type 1 response is mounted and pro-inflammatory cytokines like IFNγ and TNF-α are released. This leads to the induction of iNOS and NO production, which is the major *Leishmania* killer molecule in the murine system. Arginine, the substrate of NO production, is modulated by both Type 1 and Type 2 responses in a manner such that the Type 1 response increases IFNγ-induced iNOS-mediated conversion of arginine to NO, whereas the Type 2 response promotes arginase induction. [29].

High splenic arginase I expression has been documented in the hamster model of progressive visceral leishmaniasis caused by *Leishmania donovani* [30]. Further, in subsequent studies, it was shown that even *L. major* infection leads to arginase I induction in macrophages, and that host arginase I induction supports *Leishmania* growth. Host arginase I is induced in both the resistant C57BL/6 and susceptible BALB/c mouse strains. However, in C57BL/6 mice, it is induced only during foot pad swelling, and in BALB/c it parallels time of infection. Specific inhibition of host arginase I by nor-NOHA treatment decreases the parasite load and delays lesion development in susceptible BALB/c mice. On the other hand, in resistant C57BL/6 mice ornithine supplementation increases the susceptibility of infection, clearly suggesting that in cutaneous leishmaniasis the host arginase pathway is hijacked by the parasite for polyamine acquisition [31]. *L. major* increases host arginase I for enhanced polyamine production, which acts as a growth factor. Further, spermidine and spermine inhibit the pro-inflammatory cytokine response of the host, and spermine even modulates immune function by inhibiting the LPS-TLR4 pathway. The inhibition of host arginase I activity has the therapeutic effect of reduced pathology and controlled *L. major* replication by decreasing polyamine synthesis [32].

Further, it has also been reported that the inhibition of *L. major*–encoded arginase controls parasite growth. This growth inhibition is caused by decreasing ornithine availability for polyamine synthesis and not by decreasing the host NO response. It was also observed that increasing parasite arginase activity by IL-4 induction further promotes *Leishmania* growth [33]. It interesting to note here that simultaneous inhibition of both the *L. major*–encoded arginase and the host arginase I did not result in an increase in the nitrite levels or in the Type 1 immune response of the host [32], [33]. Polyamine biosynthesis in *Leishmania* occurs by the arginase-ODC pathway only. Consequently, Δ*arg L. major* are auxotrophic for polyamines. However, *L. major* lacking arginase retains infectivity in the susceptible BALB/c mouse strain. This clearly indicates that arginase-deficient *Leishmania* can survive in mice by salvaging the polyamines synthesized by the host. However, the pathology in the Δ*arg L. major* infection emerged less rapidly than in the wild-type infection. [34].

Strikingly, the active role played by *Leishmania* arginase in diverting arginine away from the iNOS pathway was demonstrated by the increased host NO response to Δ*arg Leishmania mexicana* infection in mice. The Δ*arg L. mexicana* infection led to an enhanced Type 1–associated IFNγ response as well. This led to a significant growth attenuation of Δ*arg L. mexicana* in mice [35]. This difference in survival between *L. major* and *L. mexicana* arginase knockouts can be attributed to the varied roles played by parasite-encoded arginase in the pathogenesis of different *Leishmania* species. There is also the possibility of the existence of an alternate arginase of *L. major.* Further, *L. major* might have an enhanced capacity to acquire host polyamines to support its growth in mice.

Host NO response is essential also in the control of *Toxoplasma gondii* infection [36], [37]. To circumvent this NO-mediated killing, *T. gondii* induces host arginase I protein expression in a STAT6-independent manner within 1 hour of infection. In accordance with the induction, mice lacking arginase I showed a survival advantage over control mice during experimental toxoplasmosis. Mice lacking arginase I did not lose weight and did not show any sign of toxoplasmosis, unlike the control mice [19]. Although the chief mechanism for host survival in *T. gondii* infection is attributed to a decrease in NO production, there might be additional functions of arginase in *Toxoplasma* infection.

Arginase is further involved in the pathogenesis of *Schistosoma mansoni*. It regulates the granulomatous pathology of schistosomiasis in vivo. In infected lung tissues, arginase activity is induced by scistosome eggs. Most of the arginase activity was derived from the host arginase I isoforms as observed by both RNA and protein data [38]. Mice carrying *S. mansoni* infection further showed a heightened arginase I expression in resident peritoneal macrophages. In the same study, a 10-fold higher level of circulating ornithine-derived polyamines were found in infected mice when compared to the control group [39]. This is of pathological significance as parasitic helminthes are known to depend on their host for uptake and interconversion of polyamines. The effect of arginase inhibition has not been tested on *S. mansoni* growth. However, an inhibition of the ODC enzyme by DFMO administered in drinking water after 5 weeks post-infection increases granuloma size and hepatic fibrosis in mice. This is explained by the enhanced bioavailability of L-ornithine in the absence of ODC towards ornithine amino transferase (OAT) for proline synthesis. Hence, it can be indirectly concluded from here that *Schistosoma*-mediated arginase I induction helps the parasite by increasing the available proline for increased collagen deposition [38]. However, direct proof can be obtained only after the development of a specific OAT inhibitor. In *Schistosoma*-infected livers, proteomic study shows that the overall abundance of arginase I protein is equal to that observed in uninfected livers. This data is consistent with various previous findings wherein arginase I is increased in the granuloma but not in the parenchyma [40].

*Schistosoma S. mansoni* expresses its own endogenous arginase as well. Although there are structural differences between the host and *Schistosoma* arginase, they are both functionally similar. Arginase activity increases in case of *Schistosoma* invasion of the host skin, and this increase is directly attributable to the *Schistosoma* endogenous arginase. It is probable that *Schistosoma* attempts to control toxic NO production by the resident innate immune cells in the skin through arginase. However, the exact role of the parasite arginase in *Schistosoma* pathogenesis has to be further elucidated [41].

### Survival Advantage Conferred by Arginase to Other Pathogens

The opportunistic fungal pathogen *Candida albicans* is part of the normal microflora but can cause systemic infection in immune-compromised individuals when it reaches the bloodstream. In order to escape from macrophages after being ingested, *Candida* employs a very fascinating strategy of inducing its own intracellular arginase (Car1p) and urea amidolyase to achieve hyphal switching. Once inside the macrophages, *Candida* rapidly upregulates its arginine biosynthetic genes. Arginine is further metabolized to ornithine and urea by arginase. The resulting urea is degraded to CO_2_ and NH_3_ by urea amidolyase (Dur1, 2p). CO_2_ further activates adenyl cyclase and the cAMP-dependent protein kinase A pathway, thereby activating Efg1p, which triggers the yeast-to-hypha switch of *Candida* inside macrophages, enabling its release. In addition, *Candida* induces two other endogenous arginases that are secreted out. These extracellular arginases may provide a survival benefit to *Candida* by reducing nitrosative stress via quenching the iNOS substrate arginine [42].

It is further reported that in hepatitis C virus (HCV) infection, arginase I mRNA and protein expression is elevated. siRNA-mediated inhibition of arginase I leads to the inability of HCV to stimulate hepatocellular growth. Arginase inhibition also increased NO-mediated cell death. Hence, arginase I plays a very significant role in HCV-mediated hepatocellular growth and survival [43].

### Spatial Localization of Arginases

Mammalian arginase I has a cytoplasmic localization, whereas mammalian arginase II is present in the mitochondrial matrix [2]. These two arginases can access the cytoplasmic arginine pool and modulate iNOS function by means of substrate quenching. However, this brings us to an intriguing question about how pathogenic arginases that are intracellular and not secreted outside the cytosol can get access to the host arginine pool. It is well documented that *Schistosoma* arginase is localized to the head of the organism and is not secreted upon infection [41], nor is the *H. pylori* arginase, which is again intracellular [44], or the *Leishmania* arginase, which contains a peroxisomal targeting signal (PTS-1) that directs it to the glycosome, an organelle unique to *Leishmania* that again is not secreted out [45]. The answer would probably lie in the unique ability of the pathogens to recruit the host arginine transporters to their vacuoles to access the cytoplasmic pool and also to utilize their own endogenous arginine uptake systems. It is hypothesized for *Leishmania* that it recruits host mCAT2B transporters to its parasitophorous vacuole [29]. It has been further reported that the *L. donovani* promastigote uses its own arginine permease, LdAAP3, to transport arginine across its membrane [46]. Mycobacteria infection also upregulates host arginine transport and utilizes this host-derived amino acid for its own benefit instead of synthesizing its own [18]. *Helicobacter* is known to possess its own arginine transport protein, RocE, to uptake arginine present in the extracellular milieu [44]. Further, the recruitment of the host arginine transporter mCAT1 to *Salmonella*–containing vacuoles and involvement of ArgT, the arginine permease of *Salmonella* in arginine uptake from host cytosol, has also been observed (unpublished data, P. Das, A. Lahiri, Ay. Lahiri, D. Chakravortty, et al.). Pathogens employ this clever strategy of channeling the host arginine pool to the intracellular pathogen-containing vacuole, and then uptake by its own arginine transporters inside the cytosol of the pathogen. Thus, pathogen-encoded arginases can modulate iNOS activity irrespective of their spatial localization by modulating the cellular distribution of arginine.

Taken together, it is evident that several intracellular pathogens, such as *H. pylori, Salmonella* Typhimurium, and *M. tuberculosis*, survive nitrosative stress by inducing the counteractive enzyme of iNOS, arginase, inside the host macrophages. Arginase promotes *Toxoplasma* and HCV infection as well by providing protection from host-induced NO stress. However, it is interesting to note that in the case of intracellular survival of parasites like *Trypanosoma*, *Leishmania*, and *Schistosoma*, arginase offers a survival advantage mainly by a polyamine-dependent and NO-independent mechanism. Arginase comes into play even in the establishment of fungal infections. *Candida* escape from host macrophages is mediated by arginase induction. Thus, arginase induction is clearly a very widespread and essential response in pathogenic infections.

## Therapeutic Implications

Targeting arginase and the polyamine biosynthetic pathways is being attempted nowadays in various diseases such as African sleeping sickness, Chagas disease, and leishmaniasis [47]. Arginase inhibition has high therapeutic value in disorders due to impaired NO production like psoriasis, septic shock, vascular diseases, airway hyper responsiveness, and rheumatoid arthritis.

Arginase isoforms regulate the availability of proline for cell proliferation and collagen deposition during diseases such as asthma and cancer. In these cases disease progression can be delayed by inhibiting arginase by nor-NOHA [48]. Although most of the work cited here deals with the mouse system, let us now consider the cases where arginase function has been targeted for therapeutic application in humans. It has been observed recently that HCV induces arginase expression in liver carcinoma, and arginase I–specific siRNA inhibited the ability of the virus to stimulate hepatocellular growth [43]. In addition, inhibition of ODC by the irreversible ODC inhibitor DFMO and the specific inhibition of spermidine biosynthesis by cyclohexylamine are routinely done in trypanosomiasis treatment [49], [50]. For the treatment of human leishmaniasis and sleeping sickness, polyamine synthesis inhibitors have been proved to be useful. Now that we know that arginase is a crucial factor for the survival of human pathogens, investigators can look more closely for new and specific inhibitors of arginase.

It has been further observed that in *Leishmania* infection in young mice, the parasite burden was higher than in the aged mice [51]. When the underlying mechanism was addressed, it was found that the young mice actually express a higher level of arginase than the older mice group, and this age-related alteration of arginase impacts the severity of *Leishmania* pathogenesis. Thus, studies in this line to understand the age-related expression of arginase in humans should be conducted prior to targeting the arginase pathway or arginine metabolism during disease.

Pathogens have evolved different strategies to escape immune responses, especially by taking advantage of the host defense mechanisms developed to cope with the invading pathogen. Here, in this review, we have summarized how a metabolic enzyme used for urea production and involved in nitrogen metabolism is hijacked by various pathogens towards their own survival. Modulation of the arginase pathway leads to decreased bactericidal NO production, increased or decreased apoptosis, and increased polyamine or proline synthesis. Each of the organisms that we discussed tries to downregulate host NO production, but the various other effects that are achieved by modulating arginase function is pathogen specific. It is tempting to propose that these effects might be used to subvert normal host cellular functions that are needed to counteract the pathogenic insult.

It should be kept in mind that arginase induction benefits the host by reducing the detrimental effect of NO and supplying polyamines for cell proliferation and proline for collagen deposition. Further, the urea cycle is an essential biochemical pathway in the host needed to clear the toxic waste product ammonia. Arginase also potentially regulates arginine-dependent immune functions such as T lymphocyte activation. Thus, any approach to treat pathogenic diseases by host arginase inhibition should be addressed with considerable caution.

## Conclusions

**Figure 2:**
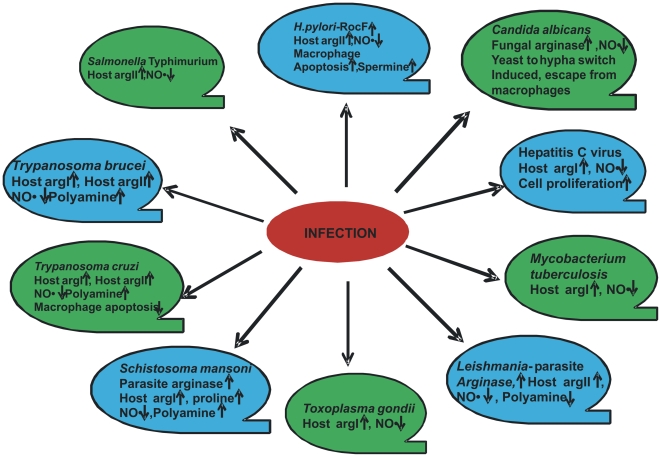
Modulation of arginase by various pathogens.

Thus, the critical interplay between the host and the pathogen to regulate arginase isoforms could determine the outcome of several infections. For example, *Staphylococcus aureus* is a pathogen carrying its own arginase and might modulate host arginase [52]. This pathway might be one of the important mechanisms of *Staphylococcus* to avoid host immune response and remains to be validated. Although considerable data have been generated in studies of the ten pathogens described in this review (Figure 2 and Table 1), many questions still remain. One of the important questions that remain to be answered is whether the pathogen gains by utilizing host arginase. How much physiological significance does this modulation have? How effective will arginase inhibition prove in any disease in human? What are the moieties of the pathogen that actually lead to arginase modulation? Taken together, arginase in pathogenesis will be a fruitful avenue of further research and will stimulate further research on both arginine metabolism and arginase function in the context of pathogenesis.

**Table 1:** Modulation of arginase by various pathogens.

Pathogen	Modified Isoforms/Bacterial Arginase	Cell Type	Possible Moiety Involved	Effect
*H. pylori*	Host argII, RocF	Macrophage,T cell; gastritis tissue	Unknown	**↓**NO°,**↑**spermine, **↑**macrophage apoptosis
*Salmonella* Typhimurium	Host argII	Macrophage, spleen; J774.1 macrophage	LPS/PAMPs	**↓**NO°
*Mycobacteria*	Host argI	Macrophage, spleen	Unknown	**↓**NO°
*Leishmania*	Host argI, parasitic arginase	Macrophage	Unknown	**↓**NO°
*Toxoplasma gondii*	Host argI	BMDM	Unknown	**↓**NO°
*Schistosoma mansoni*	Host argI, parasite arginase	Peritoneal macrophage	Unknown	**↓**NO°,**↑**polyamine
*Trypanosoma cruzi*	Host argI and II	Spleenic macrophage, cardiocyte	Cruzipain	**↓**NO°,**↑**polyamine, **↓**macrophage apoptosis
*Trypanosoma brucei*	Host argI and II	Macrophage	Unknown	**↓**NO°,**↑**polyamine
*Candida albicans*	Fungal arginase	Macrophage	Not applicable	Yeast-to-hypha switch enabling its release inside macrophage
